# Early pain-response trajectories after basivertebral nerve ablation and their relationship to 12-month clinical outcomes

**DOI:** 10.1016/j.inpm.2026.100799

**Published:** 2026-06-07

**Authors:** Charles A. Odonkor, Muhammad Uzair Siddique, Mustafa Reha Dodurgali, Sarvesh Palaniappan, Cameron Harris, Selaiman Noori, Jack Diep, Jared Rosenberg, Sudhir K. Kadian, Mannat Kadian, David W. Lee, Brian Durkin, Peter G. Whang

**Affiliations:** a Department of Orthopaedics and Rehabilitation, Division of Physiatry, Interventional Pain Medicine, Yale New Haven Hospital, 20 York Street, New Haven, CT, 06510, United States; b Department of Orthopaedics and Rehabilitation, Yale University School of Medicine, 333 Cedar Street, New Haven, CT, 06510, United States; c Boston University Chobanian and Avedisian School of Medicine, 72 East Concord St., Boston, MA, 02118, United States; d Valley Health System Graduate Medical Education, 6655 S. Cimarron Rd STE 100, Las Vegas, NV, 89113, United States; e Lakeside Spine & Pain, 1720 Mesquite Ave, Lake Havasu City, AZ, 86403, United States; f Department of Anesthesiology, Greenwich Hospital, 5 Perryridge Road, 2nd Floor, Greenwich, CT, 06830, United States; g Department of Anesthesiology and Pain Medicine, Northeast Medical Group, 194 Howard Street, New London, CT, 06320, United States; h University of Connecticut, 2131 Hillside Road, Storrs, CT, 06269, United States; i Department of Physical Medicine and Rehabilitation, University of California, Irvine, 101 the City Dr S Building 3, Orange, CA, 92868, United States; j New York Spine and Pain Specialists, 5316 Nesconset Highway, Suite 100, Port Jefferson Station, NY, 11776, United States; k Department of Orthopaedics and Rehabilitation, Division of Spine Surgery, Yale University School of Medicine, 20 York Street, New Haven, CT, 06510, United States

**Keywords:** Basivertebral nerve ablation, Vertebrogenic low back pain, Pain-response trajectory early response, Healthcare utilization, Longitudinal outcomes

## Abstract

**Background:**

Basivertebral nerve ablation (BVNA) is an established treatment for vertebrogenic low back pain, but the relationship between early post-procedural response and longer-term outcomes remains unclear.

**Objective:**

To evaluate whether early pain-response trajectories following BVNA were associated with 12-month clinical outcomes and to identify a clinically useful time point for reassessment.

**Methods:**

This prospective multicenter observational cohort study included 140 patients with MRI-confirmed Modic type 1 or 2 changes treated with BVNA between April 2024 and April 2025. Patients reported weekly percent pain relief through 8 weeks, followed by assessments at 3, 6, and 12 months. The primary outcome was ≥50% pain relief at 12 months. Discriminative performance of Week 3 and Week 6 responses was assessed using sensitivity, specificity, likelihood ratios, and receiver operating characteristic analysis. Time to first ≥50% relief was evaluated using Kaplan–Meier methods. Secondary outcomes included changes in Numeric Rating Scale (NRS), Oswestry Disability Index (ODI), Patient Global Impression of Change (PGIC), and post-procedural healthcare utilization.

**Results:**

At 12 months, 82.1% achieved ≥50% pain relief. The mean NRS improved by 4.3 points and the mean ODI improved by 34 points, with 84.3% and 94.3% achieving Minimally Clinically Important Difference (MCID) thresholds, respectively. The Week 3 response was linked to increased likelihood of 12-month success (LR^+^ 2.13; LR^−^ 0.25). Discrimination of long-term outcomes was greater at Week 6 when analyzed continuously (AUC 0.96), with an optimal threshold of approximately 38% pain relief. All patients who achieved ≥30% pain relief by Week 6 also achieved clinically meaningful improvement at 12 months, whereas only 50% of those without early improvement achieved this threshold**.** Most 12-month responders achieved ≥50% relief within 4–6 weeks. Early improvement also corresponded to greater functional recovery and reduced subsequent spine-related interventions.

**Conclusion:**

Early pain-response trajectories following BVNA were associated with 12-month outcomes. Clinically meaningful submaximal improvement (≥30%) identified patients who attained durable benefit, whereas absence of early improvement was linked to a lower likelihood of long-term success. These findings suggest that meaningful response signals may emerge before conventional ≥50% responder thresholds are reached, providing a more nuanced framework for interpreting early post-procedural response.

## 1 Introduction

Chronic low back pain (CLBP) remains one of the leading causes of disability worldwide and is projected to affect over 840 million people globally by 2050 [1]. Among patients with persistent axial low back pain, a distinct subgroup experiences vertebrogenic pain arising from damaged and inflamed vertebral endplates innervated by the basivertebral nerve (BVN). Magnetic resonance imaging (MRI) evidence of Modic type 1 or type 2 changes has emerged as a reproducible imaging biomarker of this pain phenotype and is associated with greater pain intensity, disability, and healthcare utilization [[2], [3], [4]].

Intraosseous basivertebral nerve ablation (BVNA) has been shown to be an effective and increasingly accepted treatment for vertebrogenic CLBP [[2], [3], [4], [5], [6], [7], [8], [9], [10]]. Randomized sham-controlled trials and subsequent prospective cohort studies have consistently reported substantial improvements in pain and function following BVNA, with approximately 65–75% of patients achieving clinically meaningful pain relief and durable reductions in disability at 12 months [[5], [6], [7], [8]]. Importantly, these improvements appear to persist over time, with treatment benefits maintained through 2-, 5-, and more recently 7-year follow-up in long-term extension studies [[5], [6], [7], [8], [9], [10]].

Although the first generation of BVNA literature has established that the procedure provides meaningful average improvements at 3, 6, and 12 months, these studies provide limited guidance regarding how clinicians should interpret the therapeutic response during the early post-procedure period [[5], [6], [7], [8], [9], [10], [11]]. In routine practice, patients frequently ask whether the procedure is “working” within the first several weeks after treatment. Some patients report rapid and substantial improvement within days to weeks [11] whereas others improve more gradually over several months. A third group experiences little early improvement and subsequently wonders whether the procedure has failed or whether additional evaluation and treatment are warranted due to their lack of response [11]. At present, there is no evidence-based time point at which a lack of early improvement should prompt reconsideration of the diagnosis, reassessment of competing pain generators, or discussion of additional interventions.

Our recent prospective cohort study was the first to characterize the week-by-week timing of pain and functional improvement following BVNA [11]. In this single-center cohort, most patients experienced meaningful pain relief within 3–6 weeks, and this early improvement appeared to remain stable through 24 weeks. However, that study was primarily descriptive and specifically addressed the question of when improvement occurs following BVNA; it did not determine whether the pattern and timing of early improvement delineated long-term success [11].

The current study was designed to address this distinct and clinically actionable question. Rather than simply describing the average timing of recovery, this multicenter 12-month analysis evaluates the relationship between early pain-response trajectories and long-term clinical outcomes. Specifically, we sought to determine whether the responses at Weeks 3 and 6, the overall weekly trajectory during the first 8 weeks, and the time to first clinically meaningful relief were aligned with durable response.

There is a biologic rationale for this association [[12], [13], [14], [15], [16], [17]]. BVNA directly interrupts nociceptive signaling transmitted by the BVN from inflamed vertebral endplates. Patients who experience early improvement may therefore represent those in whom the primary pain generator has been accurately identified and effectively treated by this procedure. Conversely, the absence of an early benefit may suggest the presence of persistent contributions from alternative or overlapping pain generators such as facetogenic, sacroiliac, myofascial, or radicular sources. At the same time, a delayed response remains biologically plausible because the downstream effects of BVNA may extend beyond immediate denervation to include gradual resolution of endplate inflammation, altered nociceptive sensitization through modulation of C-fiber-mediated spinal pathways, normalization of peripheral and central sensitization, as well as potentially long-term bone remodeling and endplate healing [[17], [18], [19], [20], [21], [22]].

However, most mechanistic evidence supporting delayed improvement is extrapolated from pulsed radiofrequency and dorsal root ganglion studies rather than BVNA data specifically [[18], [19], [20], [21], [22], [23]]. Direct evidence for post-BVNA bone remodeling remains limited. One small imaging study found no structural bone abnormalities on 3-month follow-up CT scans after BVNA, although that investigation was not designed to evaluate whether subtle endplate healing or osseous remodeling contributes to delayed clinical improvement [17]. These mechanisms may help explain why a subset of patients experience slower improvement despite ultimately deriving durable benefit from this procedure.

Existing studies evaluating determinants of BVNA success have largely focused on baseline demographic and clinical variables including age, pain duration, disability, pain location, and provoking activities [[11], [12], [13]]. Although these factors may modestly influence outcomes, they provide limited discrimination and may be less clinically useful than observing the patient's actual response after treatment has occurred. In other interventional pain procedures, early treatment response often correlates with longer-term success more accurately than baseline characteristics alone [24,25] but whether this is also true after BVNA remains unknown. Accordingly, the objective of this study was to determine whether early pain-response trajectories following BVNA were associated with 12-month clinical outcomes.

## 2 Methods

### 2.1 Study design and oversight

This prospective, multicenter observational cohort study evaluated the relationship between trajectory of early pain relief following basivertebral nerve ablation (BVNA) and 12-month clinical outcomes. The study was conducted across three participating centers, including the original academic center and two additional affiliated spine and pain practices. All sites prospectively collected data using the same standardized protocol, follow-up schedule, and outcome measures. The current study represents an expansion of our previously reported prospective cohort, which characterized the week-by-week timing of symptom improvement through 24 weeks. In contrast, the present analysis was designed to address a different and more clinically actionable question: whether early response trajectory is linked to long-term success at 12 months and whether a clinically useful time point for reassessment can be identified.

Eligible patients were enrolled consecutively between April 2024 and April 2025. They were followed from baseline through 12 months after BVNA. Assessments were obtained weekly during the first 8 weeks after treatment and then again at 3, 6, and 12 months. Data collection occurred through in-person visits, telephone follow-up, or secure electronic surveys. The study was conducted in accordance with the ethical principles of the Declaration of Helsinki. All participants provided written informed consent before enrollment which had received approval from the Yale University School of Medicine Institutional Review Board (IRB #2000040858). No industry funding or sponsorship supported the BVNA procedures, follow-up evaluations, or related clinical care. All study-related activities were financed through standard clinical care pathways and institutional research support.

### 2.2 Participants

Patients were eligible if they had chronic axial low back pain for at least 6 months despite having completed guideline-directed conservative treatment; demonstrated MRI-confirmed Modic type 1 and/or type 2 changes at the vertebral levels selected for BVNA; and had a clinical presentation consistent with vertebrogenic pain including midline or paramedian low back pain without predominant radicular symptoms [2]. All patients underwent BVNA at one or more clinically indicated lumbar vertebral levels. Patients were excluded if they had an active infection, malignancy, fracture, or major structural deformity precluding BVNA; were unable to complete follow-up assessments; or had significant psychiatric or medical comorbidities likely to confound outcome assessment.

### 2.3 BVNA procedure

BVNA was performed under fluoroscopic guidance using the Intracept® System (Boston Scientific, MA, USA) according to standard clinical practice. All procedures were performed by fellowship-trained interventional pain physicians, interventional radiologists, and surgeons at each participating site. The target vertebral levels were selected based on the presence of Modic changes and clinical correlation. Because the technical details of the procedure have been described previously [11], only a brief summary is provided here. Following transpedicular access, the radiofrequency probe was advanced to the anticipated location of the basivertebral nerve within the posterior vertebral body and thermal ablation was performed according to manufacturer-recommended parameters. Additional details regarding probe targeting, lesion creation, and peri-procedural management have been reported in our prior publication [11].

### 2.4 Outcome measures

The primary outcome was durable clinical response at 12 months, defined as ≥50% pain relief relative to baseline. Secondary outcomes included percent pain relief at each weekly and monthly follow-up visit, time to the initial report ≥50% pain relief, Numeric Rating Scale (NRS) pain intensity, Oswestry Disability Index (ODI), Patient Global Impression of Change (PGIC), achievement of clinically meaningful ODI improvement, and changes in post-BVNA spine-related procedural utilization. Clinically meaningful pain improvement was defined as a ≥3-point reduction in NRS, clinically meaningful functional improvement was defined as a ≥15-point reduction in ODI [10,11], and clinically meaningful global improvement was defined as PGIC ≥5, whereas PGIC ≥6 represented patients reporting that they were “much improved” or “very much improved.” The primary outcome, exposures (early response measures), and covariates (demographic, clinical, and procedural variables) were defined a priori based on clinical relevance and prior literature.

### 2.5 Early response and trajectory definitions

To distinguish different clinical patterns of response, patients were classified into four pre-specified trajectory groups based on the percent pain relief reported at Week 6 and 12 months, as follows: a. Early sustained responders: ≥50% pain relief at Week 6 and 12 months; b. Delayed responders: <50% at Week 6 but ≥50% at 12 months; c. Transient responders: ≥50% at Week 6 but <50% at 12 months; and d. Persistent nonresponders: <50% at both timepoints.

Week 3 response was also analyzed as an exploratory early signal, not as a defining criterion for trajectory classification in this study. Prior analyses suggested that the 3–6-week window after BVNA may represent an inflection point in the response trajectory [11], thus, these time points were chosen to evaluate whether the degree and timing of early improvement would be associated with 12-month outcomes.

### 2.6 Bias minimization

Several measures were incorporated to reduce the potential for bias inherent to an observational study design. Potential sources of bias included selection bias, measurement bias from patient-reported outcomes, and confounding by indication inherent to observational procedural studies. Data collection and abstraction were standardized across participating sites, and all primary and secondary analyses were pre-defined before data collection and statistical testing. Multivariable models were used to account for clinically relevant covariates, and study sites were included to address potential center-level variation. Additional sensitivity and exploratory analyses were performed to assess the robustness of the findings.

### 2.7 Sample size

The sample size was determined a priori based on a prospective cohort design with consecutive patient inclusion over a defined study period, consistent with our prior work examining early response trajectories following BVNA [11]. In this context and given exploratory analyses, formal power calculations were not used to define enrollment targets, as the primary objective was to evaluate longitudinal associations between early response patterns and 12-month outcomes within a real-world population. The final cohort of 140 patients represents, to our knowledge, the largest prospective dataset to date evaluating early response trajectories following BVNA. The study cohort aligns with commonly cited sample size considerations for regression modeling relative to the number of covariates evaluated.

### 2.8 Statistical analysis

Continuous variables are reported as mean ± standard deviation or median (interquartile range) as appropriate, whereas categorical variables are presented as counts and percentages. The association of an early response with 12-month outcomes was evaluated using two complementary approaches. First, dichotomous response thresholds, defined as ≥50% pain relief at Weeks 3 and 6, were examined followed by the evaluation of continuous percent pain relief at Weeks 3 and 6. For each early response threshold, sensitivity, specificity, positive predictive value, negative predictive value, and likelihood ratios were calculated. These metrics were used to evaluate association of early response with durable 12-month response. Receiver operating characteristic (ROC) curves were constructed to evaluate the ability of continuous Week 3 and Week 6 pain relief to discriminate between 12-month responder and nonresponder **status** within the cohort and to identify the threshold that provided the greatest separation. The area under the curve values were compared between Week 3 and Week 6 analyses whereas a Kaplan–Meier analysis was performed to estimate time to first achievement of ≥50% pain relief following BVNA. Curves were stratified according to eventual 12-month responder status, and the proportion of patients who had not yet achieved ≥50% pain relief was calculated at each weekly follow-up interval.

Multivariable logistic regression was performed to identify factors independently associated with 12-month response. The primary analysis focused on the association between Week 6 response and 12-month outcome, whereas secondary and exploratory analyses were interpreted accordingly. Candidate covariates included Week 6 response status, age, sex, body mass index (BMI), pain duration, baseline NRS, baseline ODI, number of vertebral levels treated, study site, and prior failed spine-related procedures. At some centers, because insurance restrictions sometimes limited the number of levels treated per procedure despite the presence of additional Modic-positive levels, the number of levels treated was interpreted in the context of underlying disease burden. To account for this, the ratio of treated levels to total Modic-positive levels was examined in exploratory analyses. In addition, the total number of prior spine-related procedures was substituted for the binary history of failed prior procedures in order to determine whether procedural burden was independently associated with long-term outcomes. Secondary and exploratory analyses were prespecified. No imputation was performed for missing data as follow-up completeness was high. To account for the multiple correlated comparisons across weekly thresholds and secondary analyses, false discovery rate adjustment was performed using the Benjamini–Hochberg procedure, which is less conservative and more commonly used for correlated comparisons in repeated-measures settings, and more statistically appropriate than Bonferroni correction. Statistical analyses were conducted using R version 4.3.2 (R Foundation for Statistical Computing, Vienna, Austria). A two-sided p value < 0.05 after correction was considered statistically significant.

## 3 Results

### 3.1 Participant characteristics and baseline comparisons

**Fig. 1:**
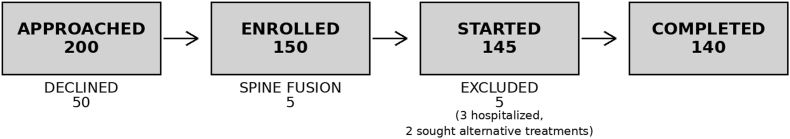
*Study enrollment and completion flow.* Of the 200 patients approached, 150 were enrolled. Five patients underwent spine fusion prior to treatment and were excluded. A total of 145 initiated the study, and 5 were excluded prior to BVNA (hospitalization, n = 3; alternative treatment, n = 2), resulting in 140 patients completing treatment and included in the final analysis.

A total of 140 patients who underwent BVNA were included in the final analysis (Table 1). Fig. 1 displays the enrollment and study completion flow. Of the 200 patients approached for participation, 150 were enrolled; 5 proceeded to spine fusion prior to treatment and were therefore excluded. A total of 145 patients initiated the study, and 5 were excluded prior to BVNA due to pre-procedural hospitalization (n = 3) or pursuit of alternative treatments (n = 2). Ultimately, 140 patients completed BVNA treatment with 12-month follow-up and were included in the final analysis. Overall, 115 patients (82.1%) achieved the primary outcome of ≥50% pain relief at 12 months, whereas 25 (17.9%) did not reach this threshold.

**Table 1:** Baseline characteristics of the study cohort stratified by 12-month response status.

Characteristic	Overall (N = 140)	12-Month Responders (n = 115)	12-Month Nonresponders (n = 25)	P value
Age, years	64.1 ± 13.2	64.6 ± 12.8	61.8 ± 14.9	0.318
Female sex, n (%)	79 (56.4)	64 (55.7)	15 (60.0)	0.827
BMI, kg/m^2^	31.8 ± 6.7	31.4 ± 6.5	33.8 ± 7.5	0.120
Baseline NRS	7.8 ± 1.4	7.7 ± 1.4	8.2 ± 1.5	0.094
Baseline disability, %	64.7 ± 15.1	63.8 ± 15.3	68.9 ± 13.5	0.118
Chronic pain duration, y	8.4 ± 5.3	8.0 ± 5.1	10.1 ± 5.9	0.081
Levels treated, n	3.4 ± 1.1	3.8 ± 1.2	3.3 ± 1.0	0.046
Modic levels present, n	3.9 ± 1.2	3.8 ± 1.1	4.0 ± 1.3	0.080
Lesion strategy				0.412
All 7-min lesions	32 (22.9)	27 (23.5)	5 (20.0)	
All 15-min lesions	51 (36.4)	40 (34.8)	11 (44.0)	
Mixed 7- and 15-min lesions	57 (40.7)	48 (41.7)	9 (36.0)	

Values are presented as mean ± standard deviation unless otherwise indicated. P values were calculated using the Mann–Whitney *U* test for continuous variables and Fisher exact or χ^2^ testing for categorical variables.

Baseline characteristics stratified by 12-month response status are summarized in Table 1. In general, responders and nonresponders were well-balanced across most demographic and clinical characteristics, supporting baseline comparability between groups. Age, sex, BMI, baseline pain severity, baseline disability, chronic pain duration, number of Modic levels, and lesion strategy did not significantly differ between groups. Responders and nonresponders were similar in age (64.6 ± 12.8 vs 61.8 ± 14.9 years, p = 0.318), sex distribution (55.7% vs 60.0% female, p = 0.827), BMI (31.4 ± 6.5 vs 33.8 ± 7.5 kg/m^2^, p = 0.120), and baseline NRS pain score (7.7 ± 1.4 vs 8.2 ± 1.5, p = 0.094). Baseline disability also did not differ significantly between groups (63.8 ± 15.3% vs 68.9 ± 13.5%, p = 0.118).

Chronic pain duration (10.1 ± 5.9 vs 8.0 ± 5.1 years, p = 0.081), Modic-positive levels (4.0 ± 1.3 vs 3.8 ± 1.1, p = 0.080), and baseline pain did not differ significantly between groups. The only baseline characteristic that differed significantly between groups was the number of vertebral levels treated (3.8 ± 1.2 vs 3.3 ± 1.0, p = 0.046); however, this variable should be interpreted cautiously given potential mismatch between treated levels and underlying Modic-positive disease burden.

Importantly, lesion strategy was not associated with 12-month outcomes. Rates of response were similar among patients treated with exclusively 7-min lesions, exclusively 15-min lesions, or mixed lesion-duration strategies (p = 0.412), suggesting that the associated findings observed in the present study were not driven by differences in ablation duration.

### 3.2 Long-term 12-month pain and functional outcomes

At 12 months, 115 of 140 patients (82.1%) achieved ≥50% pain relief following BVNA. The mean pain intensity improved from 7.8 ± 1.4 at baseline to approximately 3.5 at 12 months, corresponding to a mean reduction of 4.3 points; similarly, disability improved from 64.7 ± 15.1% to approximately 30 ± 4.5%, corresponding to a mean ODI reduction of 34 points (Table 2). These observed changes met established MCID thresholds for clinically meaningful improvement (i.e. ≥3 points for NRS and ≥12 points for ODI). At 12 months, 118 of 140 patients (84.3%) achieved the NRS MCID of at least a 3-point reduction and 132 of 140 patients (94.3%) achieved the ODI MCID of at least a 12-point improvement (Table 2).

**Table 2:** Long-term 12-month pain and functional outcomes.

Outcome	Baseline Mean ± SD	12-Month Mean ± SD	Mean Change	MCID Threshold	Patients Meeting MCID, n (%)
NRS pain score	7.8 ± 1.4	3.5 ± 2.4	−4.3 points	≥3-point reduction	118/140 (84.3%)
ODI disability score	64.7 ± 15.1	30.0 ± 4.5	−34.7 points	≥12-point improvement	132/140 (94.3%)

Unlike the original prospective study [11] which focused primarily on describing the week-by-week trajectory of symptom improvement through 24 weeks, the present companion cohort was designed to determine whether early improvement is linked to long-term benefits at 12 months. Accordingly, subsequent analyses focused on the clinical value of early response rather than the descriptive trajectory itself.

### 3.3 Week 3 and week 6 response analyses

The discriminatory value of early response is summarized in Table 3. Patients who achieved ≥50% pain relief by Week 3 were substantially more likely to remain responders at 12 months. Week 3 response was associated with a sensitivity of 0.826 and specificity of 0.600 for 12-month success, with a positive predictive value of 0.905 and a negative predictive value of 0.429. Week 3 response was also associated with a positive likelihood ratio (LR+) of 2.13 (95% CI 1.31–3.46) and a negative likelihood ratio (LR−) of 0.25 (95% CI 0.14–0.42). Thus, meaningful pain relief within the first 3 weeks corresponded to an increased likelihood of durable benefit in this cohort.

**Table 3:** Likelihood ratios of early response at weeks 3 and 6 for 12-month response following basivertebral nerve ablation.

Early Response	TP	FP	FN	TN	Sensitivity	Specificity	PPV	NPV	LR+ (95% CI)	LR− (95% CI)
Week 3 response (≥50% relief)	95	10	20	15	0.826	0.600	0.905	0.429	2.13 (1.31–3.46)	0.25 (0.14–0.42)
Week 6 response (≥50% relief)	111	24	4	1	0.965	0.040	0.822	0.200	1.01 (0.95–1.08)	0.88 (0.15–5.07)

Abbreviations: FN, false negative; FP, false positive; LR+, positive likelihood ratio; LR−, negative likelihood ratio; NPV, negative predictive value; PPV, positive predictive value; TN, true negative; TP, true positive.

**Fig. 2:**
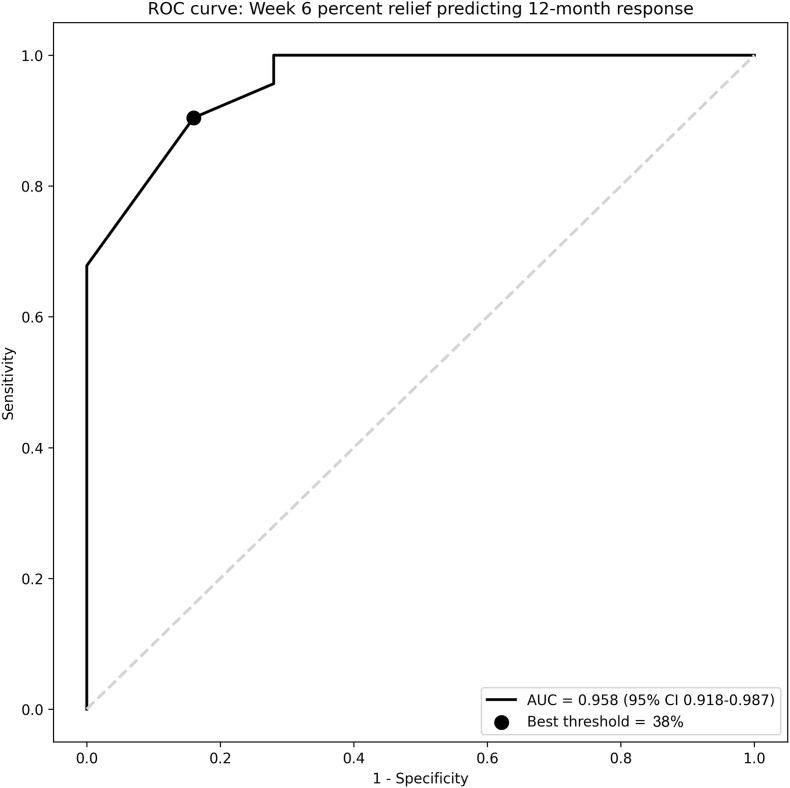
*Receiver operating characteristic curve evaluating the ability of continuous percent pain relief at Week 6 to identify* 12-month *response*. The area under the curve was 0.958 (95% CI 0.918–0.987), indicating good discriminative ability. The optimal Week 6 threshold identified by the Youden index was approximately 38% pain relief, yielding a sensitivity of 0.87 and specificity of 0.68 for detecting durable 12-month response.

In contrast, the simple binary Week 6 threshold of ≥50% pain relief performed less well because nearly all patients crossed this threshold by Week 6. Although binary ≥50% response at Week 6 had limited specificity because most patients crossed this threshold, continuous Week 6 pain relief demonstrated strong discrimination, with the most informative threshold occurring below the conventional responder cutoff. When Week 6 pain relief was analyzed as a continuous variable, greater discriminatory performance was observed. The receiver operating characteristic curve demonstrated good discrimination for 12-month response, with an area under the curve of 0.96 (95% CI 0.92–0.99, Fig. 2). The optimal Week 6 threshold identified by the Youden index was approximately 38% pain relief, which yielded a sensitivity of 0.87 and specificity of 0.68 for identifying durable 12-month response.

Thus, a key finding was not whether patients had achieved a full ≥50% response by Week 6, but whether they demonstrated approximately 35–40% early improvement.

### 3.4 Longitudinal response patterns

**Fig. 3:**
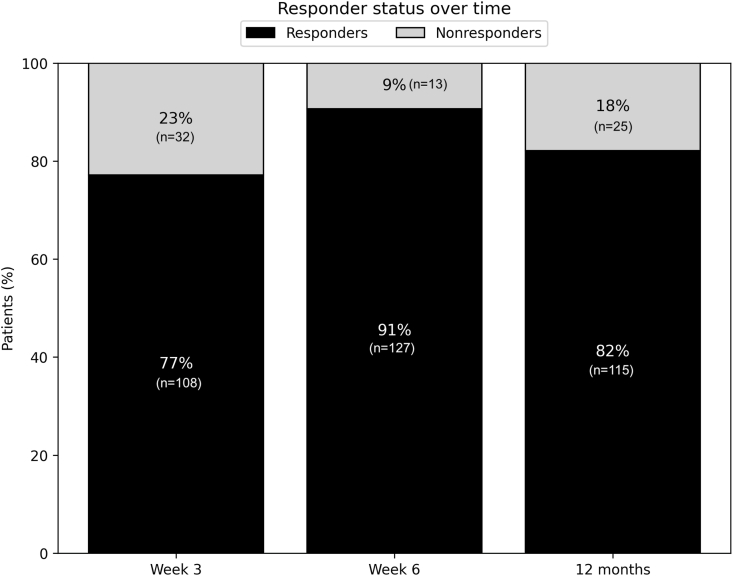
*Stacked bar graph showing the proportion of patients achieving ≥50% pain relief at Week 3, Week 6, and* 12 months *following BVNA*. Black bars indicate responders and light gray bars indicate nonresponders. The proportion of responders increased from 77% (108/140) at Week 3 to 91% (127/140) at Week 6, with 82% (115/140) maintaining response at 12 months.

Although Week 3 response provided an early signal of treatment trajectory, formal classification of response patterns was based on Week 6 status. Among patients who had not achieved ≥50% pain relief by Week 3 (n = 32), 17 (53.1%) subsequently met responder criteria at 12 months. The remaining 15 accounted for 60% of the 25 nonresponders observed at 12 months (Fig. 3). Importantly, among the 127 patients who were responders at Week 6, 115 maintained responses at 12 months, supporting the role of Week 6 as a more stable inflection point for trajectory classification.

Patients who had lower Week 6 improvement demonstrated reduced likelihood of durable response, but binary ≥50% Week 6 responder status had limited specificity because most patients crossed this threshold by that time.

### 3.5 Response trajectories stratified by week 6 status

**Fig. 4:**
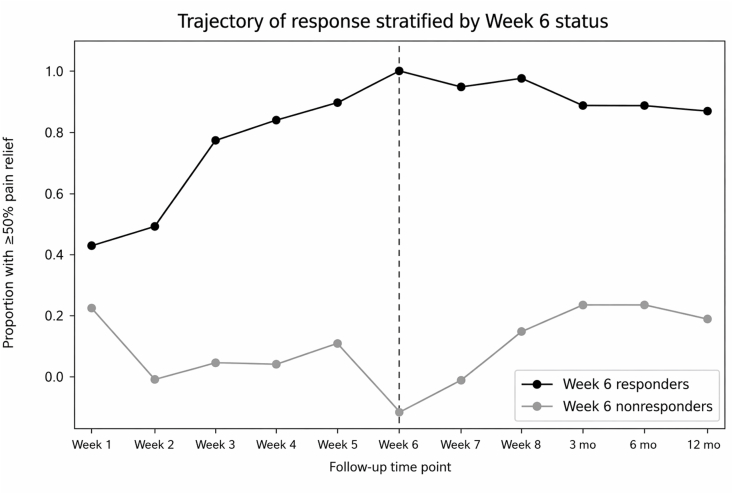
*Longitudinal Response Trajectories Stratified by Week 6 Responder Status.* The line graph shows the proportion of patients achieving ≥50% pain relief over time, stratified by Week 6 responder status. The dashed vertical line indicates the Week 6 assessment. Patients who were responders at Week 6 maintained high response rates through 12 months, whereas Week 6 nonresponders remained below the responder threshold throughout follow-up.

As shown in Fig. 4, patients classified by Week 6 response status demonstrated early and sustained separation in outcomes beginning as early as Week 3. Among Week 6 responders (≥50% pain relief), the proportion of responders increased from 77% at Week 3 to 100% at Week 6 and remained high through 12 months (87%), indicating durable maintenance of treatment effect. In contrast, Week 6 nonresponders (<50% pain relief) demonstrated persistently lower response rates over time, with substantially reduced likelihood of achieving durable benefit at 12 months.

In a secondary IMMPACT-aligned sensitivity analysis using a ≥30% pain relief threshold at Week 6 (Table 4), 122 of 140 patients (87.1%) met criteria for early clinically meaningful improvement. All of these patients (122/122, 100%) achieved ≥30% pain relief at 12 months, corresponding to a positive predictive value of 100% within this cohort. Among the 18 patients who failed to achieve ≥30% improvement by Week 6, only 9 (50.0%) subsequently achieved ≥30% pain relief at 12 months. Notably, absence of ≥30% improvement at Week 6 was linked to substantially lower likelihood of achieving clinically meaningful pain relief at 12 months, supporting the relevance of early submaximal response.

**Table 4:** **IMMPACT-Aligned Sensitivity Analysis of Week 6 Response and Detection of 12-Month Outcomes**. This analysis uses an IMMPACT-aligned threshold of ≥30% pain relief to evaluate early clinically meaningful improvement. Primary analyses in the manuscript are based on the ≥50% responder definition.

Panel A. Predictive Value of Week 6 ≥ 30% Pain Relief for 12-Month ≥30% Response						
Week 6 Threshold	Week 6 Responders, n (%)	Week 6 Nonresponders, n (%)	12-Month ≥30% Responders Among Week 6 Responders	12-Month ≥30% Responders Among Week 6 Nonresponders	Positive Predictive Value	Negative Predictive Value
≥30% pain relief	122/140 (87.1%)	18/140 (12.9%)	122/122 (100.0%)	9/18 (50.0%)	100.0%	50.0%

Panel B. Cross-Classification of Week 6 IMMPACT Response and 12-Month Outcomes		
Week 6 IMMPACT Response	n (%) of Cohort	12-Month ≥30% Responders, n/N (%)
Responders (≥30%)	122/140 (87.1%)	122/122 (100.0%)
Nonresponders (<30%)	18/140 (12.9%)	9/18 (50.0%)

Together, these findings suggest that an early signal at approximately 6 weeks emerges before patients necessarily achieve a full ≥50% response and is consistent with established IMMPACT-aligned thresholds for clinically meaningful improvement [26].

### 3.6 Time-to-response analysis

**Fig. 5:**
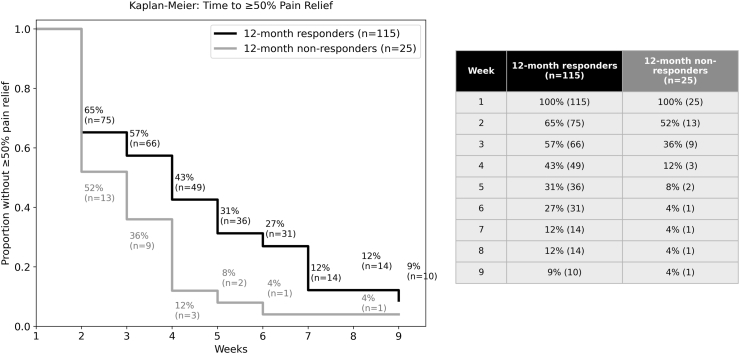
*Time to First ≥50% Pain Relief Stratified by 12-Month Response Status.* Kaplan–Meier curves show the time to first achievement of ≥50% pain relief following BVNA, stratified by ultimate 12-month response status. Black lines represent patients who were responders at 12 months and light gray lines represent nonresponders. Most eventual responders achieved ≥50% pain relief within the first 4–6 weeks, whereas relatively few eventual nonresponders ever crossed this threshold. The accompanying table displays the proportion and number of patients remaining below the ≥50% threshold at each weekly time point.

The Kaplan–Meier analysis demonstrated that patients who ultimately attained a durable 12-month response tended to experience earlier and more rapid improvement than patients who did not reach this threshold (Fig. 5). The curves separated as early as Week 2 and remained distinct throughout the entire follow-up period. Among 12-month responders, 35.0% had achieved their first episode of ≥50% pain relief by Week 3, 57.4% by Week 4, and 73.0% by Week 6. In contrast, only 13.0% of eventual nonresponders had achieved ≥50% pain relief by Week 4 and just 5.0% by Week 6. (Fig. 5). Most patients who achieved durable 12-month benefit (57–73%) reached the ≥50% threshold within 4–6 weeks. Conversely, relatively few eventual nonresponders ever crossed this threshold during the observation period. By Week 8, most eventual responders had achieved ≥50% pain relief, whereas delayed crossing of this threshold was uncommon among eventual nonresponders (Fig. 5). These findings suggest that the timing of early improvement was linked to differences in 12-month outcomes independent of the absolute degree of pain relief at any single time point.

### 3.7 Functional outcomes over time

**Fig. 6:**
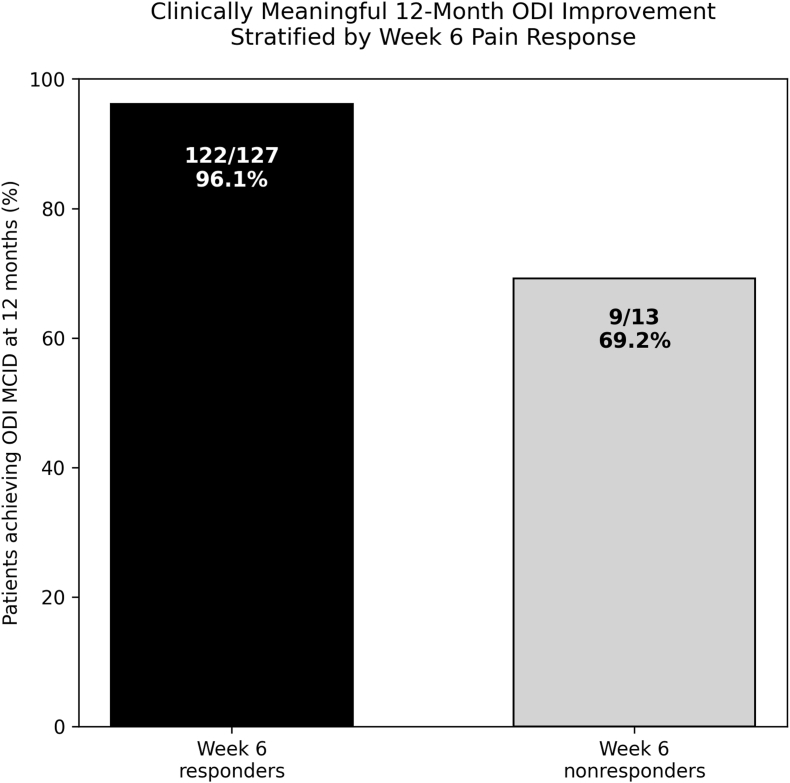
*Twelve-Month Functional Improvement Stratified by Week 6 Pain Response. The* bar graph shows the proportion of patients achieving the minimum clinically important difference (MCID) in Oswestry Disability Index (ODI) at 12 months, stratified by Week 6 pain-response status. Black bars represent Week 6 responders and light gray bars represent Week 6 nonresponders. Patients who achieved ≥50% pain relief by Week 6 were substantially more likely to achieve clinically meaningful functional improvement at 12 months (96.1% vs 69.2%).

Changes in functional disability paralleled the observed pain relief response trajectories. At baseline, 60.7% of patients had severe or crippled disability (Table 5). Over time, there was a marked shift toward lower disability categories, with most responders transitioning into the minimal or moderate disability range by 12 months (Table 5). The proportion of patients in the minimal or moderate disability categories increased from 5.7% at baseline to 74.3% at 6 weeks, 72.1% at 3 months, 75.7% at 6 months, and 74.2% at 12 months. Conversely, the proportion categorized as crippled or bed-bound declined from 60.7% at baseline to 12.8% at 6 weeks and remained below 12% through 12 months. Early improvement in pain was also linked to functional outcomes at 12 months. Patients who achieved ≥50% pain relief by Week 6 were substantially more likely to achieve clinically meaningful ODI improvement at 12 months than Week 6 nonresponders (96.1% vs 69.2%) (Fig. 6). These findings reinforce the finding that early pain reduction following BVNA was not linked to durable relief but also aligned with long-term restoration of function and ability to perform daily activities.

**Table 5:** Distribution of oswestry disability index (ODI) functional disability categories from baseline through 12 Months following basivertebral nerve ablation (BVNA).

ODI Disability Category	Baseline, n (%)	6 Weeks, n (%)	3 Months, n (%)	6 Months, n (%)	12 Months, n (%)
Minimal (0–20%)	0 (0.0)	22 (15.7)	31 (22.1)	48 (34.3)	59 (42.1)
Moderate (21–40%)	8 (5.7)	61 (43.6)	70 (50.0)	58 (41.4)	45 (32.1)
Severe (41–60%)	47 (33.6)	39 (27.9)	28 (20.0)	22 (15.7)	20 (14.3)
Crippled (61–80%)	71 (50.7)	16 (11.4)	10 (7.1)	8 (5.7)	11 (7.9)
Bed-bound (>80%)	14 (10.0)	2 (1.4)	1 (0.7)	4 (2.9)	5 (3.6)

### 3.8 Multivariable analysis of factors linked to 12-month response

The results of multivariable logistic regression are presented in Table 6. After adjustment for age, sex, BMI, chronic pain duration, treated-levels/positive Modic ratio, and prior treatment history, early improvement at Week 6 remained independently associated with durable 12-month response. Patients who achieved a Week 6 response had more than a fivefold greater odds of remaining responders at 12 months (adjusted odds ratio [aOR] 5.62, 95% CI 1.11–28.37, p = 0.037). In contrast, a history of failed prior procedures was independently associated with a lower likelihood of a durable response (aOR 0.31, 95% CI 0.11–0.89, p = 0.029).

**Table 6:** Multivariable logistic regression of 12-month response following basivertebral nerve ablation.

Variable	Adjusted Odds Ratio	95% CI	P value
Week 6 responder (≥50% relief)	5.62	1.11–28.37	0.037
Age, per year	1.01	0.97–1.05	0.634
Female sex	0.89	0.33–2.39	0.821
BMI, kg/m^2^	0.96	0.89–1.04	0.302
Chronic pain duration, per year	0.94	0.87–1.01	0.095
Levels treated/Modic ratio	0.68	0.43–1.08	0.103
Failed prior procedures	0.31	0.11–0.89	0.029

Variables entered into the model included early response status, age, sex, BMI, chronic pain duration, Treated levels/Modic ratio, and history of failed prior procedures.

There were no statistically significant associations between age, sex, BMI and the number of treated levels and 12-month outcome after multivariable adjustment. Collectively, these findings suggest that long-term BVNA outcomes may be more closely aligned with early post-procedural response trajectory and prior treatment history than with baseline demographic characteristics alone.

### 3.9 Patient global impression of change and subsequent healthcare utilization

**Fig. 7:**
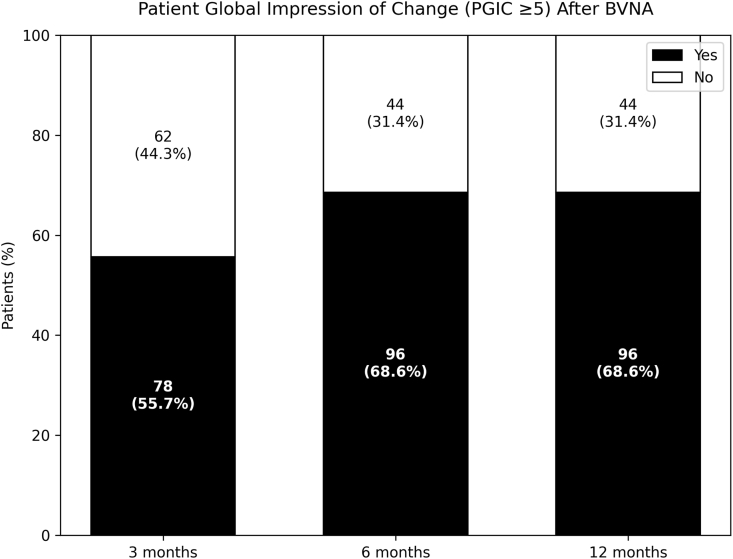
*Patient Global Impression of Change Over Time After Basivertebral Nerve Ablation*. The stacked bar graph shows the proportion of patients reporting a PGIC score ≥5 at 3, 6, and 12 months following BVNA. Black bars indicate patients reporting meaningful improvement (PGIC ≥5), and light gray bars indicate those without meaningful improvement (PGIC <5). The proportion of patients reporting meaningful global improvement increased from 55.7% at 3 months to 68.6% at both 6 and 12 months.

Patient-reported global improvement increased over time and paralleled the observed reductions in pain and healthcare utilization. At 3 months, 78 of 140 patients (55.7%) reported a PGIC score ≥5, indicating that they felt at least “moderately improved.” This proportion increased to 96 of 140 patients (68.6%) at both 6 and 12 months (Fig. 7).

These subjective improvements were accompanied by marked reductions in subsequent spine-related interventions (Table 7). The proportion of patients undergoing any spine-related injection decreased from 97.1% in the 12 months before BVNA to 49.3% in the 12 months after BVNA (difference −47.9%, 95% CI −56.4% to −39.3%; p < 0.001). Significant reductions were observed across all major injection categories including trigger point injections (72.9% to 25.7%), facet joint injections (34.3% to 8.6%), sacral injections (39.3% to 15.0%), and epidural steroid injections (28.6% to 16.4%); (all p ≤ 0.016). The completion of more advanced interventions also declined including peripheral nerve stimulation (7.9% to 1.4%) and spinal cord/dorsal root ganglion stimulation (10.0% to 5.7%). Only 5 patients (3.6%) underwent subsequent spine surgery during the 12 months after BVNA, whereas none had undergone surgery in the year prior to treatment; kyphoplasty was also uncommon and unchanged (1.4% before and after BVNA). Together, these findings suggest that the improvements in pain and function following BVNA were accompanied by durable patient-perceived benefits and reduced reliance on subsequent spine-related procedures.

**Table 7:** Spine-related healthcare utilization after BVNA.

Treatment	12 months pre-BVNA, n (%)	12 months post-BVNA, n (%)	p-value	Difference in proportions (95% CI)
Any injections*	136 (97.1%)	69 (49.3%)	<0.001	−47.9% (−56.4 to −39.3)
Trigger point injections	102 (72.9%)	36 (25.7%)	<0.001	−47.1% (−56.1 to −38.2)
Facet joint injections	48 (34.3%)	12 (8.6%)	<0.001	−25.7% (−34.9 to −16.6)
Medial branch blocks (MBB)	9 (6.4%)	0 (0.0%)	0.004	−6.4% (−10.5 to −2.4)
Sacral injections	55 (39.3%)	21 (15.0%)	<0.001	−24.3% (−31.4 to −17.2)
Epidural steroid injections	40 (28.6%)	23 (16.4%)	0.016	−12.1% (−21.3 to −3.0)
Kyphoplasty	2 (1.4%)	2 (1.4%)	1.000	0.0% (−2.8 to 2.8)
Peripheral nerve stimulation (PNS)	11 (7.9%)	2 (1.4%)	0.022	−6.4% (−11.4 to −1.5)
Spinal cord stimulation/dorsal root ganglion stimulation (SCS/DRG)	14 (10.0%)	8 (5.7%)	0.031	−4.3% (−7.6 to −0.9)
Spine surgery (fusion/laminectomy/decompression/SI joint surgery)	0 (0.0%)	5 (3.6%)	0.063	+3.6% (−0.4 to 7.5)

## 4 Discussion

In this multicenter cohort of 140 patients undergoing basivertebral nerve ablation (BVNA), most patients experienced substantial and durable improvements in pain and function through 12 months. Overall, 82.1% achieved the primary outcome of ≥50% pain relief at 12 months, accompanied by a mean 4.3-point reduction in pain intensity and a 34-point improvement in disability. More than 80% of patients met established MCID thresholds for pain improvement and more than 90% achieved clinically meaningful improvement in function. Unlike our prior prospective study [11], which described the week-by-week timing of symptom improvement through 24 weeks, the present analysis addressed a more clinically actionable question: whether the pattern and timing of early improvement are associated with long-term benefit. These findings suggest that early response patterns after BVNA are associated with 12-month outcomes.

### 4.1 Early response trajectory and durable 12-month benefit

Building on the observation that most patients improved within the first several weeks after BVNA, the current study further suggests that the pattern of that early improvement may align with durable outcomes. Patients who ultimately achieved durable 12-month benefits were more likely to have experienced earlier and more rapid improvement than those who did not. Most 12-month responders crossed the ≥50% pain-relief threshold within 4–6 weeks. In contrast, nonresponders either did not reach this threshold or achieved it later without sustained benefit.

According to this analysis, the discriminatory value of early response was greatest around Week 6. Although Week 3 response was also linked to subsequent success, the most favorable combination of sensitivity, specificity, and negative likelihood ratio occurred when Week 6 pain relief was evaluated as a continuous measure. A threshold of approximately 35–40% pain relief by Week 6 provided the greatest discrimination for durable 12-month response within the cohort. This range closely paralleled IMMPACT-aligned thresholds for clinically meaningful improvement [26].

In contrast to our prior work [11], which focused on the timing of improvement, the present study identifies when early response becomes sufficiently informative to potentially guide clinical expectations and follow-up. The current analyses show that binary ≥50% responder thresholds may perform less well early on, whereas submaximal early improvement provides greater discriminatory value.

### 4.2 Finding a clinically meaningful reassessment point

The study findings support the use of an early post-procedural time point for reassessment of patient response. Within the first few weeks, nearly three-quarters of eventual 12-month responders had already experienced their first episode of ≥50% pain relief, whereas very few eventual nonresponders had done so. Nevertheless, an absence of early meaningful improvement should not be interpreted as definitive treatment failure; specifically, a delayed-responder subgroup was observed comprised of approximately half of patients who had not yet achieved ≥50% pain relief by Week 3 but who ultimately became 12-month responders. However, delayed improvement beyond 6 weeks was uncommon in this cohort (approximately 3% of the cohort), and lack of early improvement by this time corresponded to a lower likelihood of a durable response. Patients with minimal improvement by this stage may warrant early diagnostic reassessment, including evaluation for alternative pain generators and contributing psychosocial or structural factors. Although some patients may demonstrate delayed improvement, persistent lack of response over time should prompt consideration of adjunctive or alternative treatment strategies.

### 4.3 Submaximal early improvement May provide additional insight before conventional responder thresholds are reached

An important finding of the present study is that submaximal early improvement may provide clinical insight before patients reach conventional ≥50% responder status. Using the primary ≥50% responder definition, early response by Week 6 was associated with durable 12-month benefit. In a secondary IMMPACT-aligned sensitivity analysis, achievement of ≥30% pain relief at Week 6 corresponded to sustained clinically meaningful improvement at 12 months. Among those who did not reach this threshold, 50% ultimately achieved ≥30% pain relief. These findings suggest that early response signals may emerge before patients reach conventional ≥50% responder thresholds. Rather than redefining success, the ≥30% threshold may provide a clinically interpretable marker of early meaningful improvement that precedes full responder status [26]. In this context, early submaximal improvement appears to reflect a trajectory toward durable benefit, whereas an absence of such improvement identifies a subgroup with substantially lower probability of long-term success.

From a practical standpoint, these findings may help inform the timing of reassessment and treatment decisions. Clinicians should avoid concluding that BVNA has failed within the first several weeks after the procedure but at the same time, prolonged persistence of minimal or absent improvement beyond approximately 6 weeks may indicate a lower probability of eventual durable benefit and a need for diagnostic reevaluation.

### 4.4 Exceptions to the rule: delayed responders and transient early responders

The current study also identified clinically distinct response trajectories that were not captured in the prior manuscript [11]. Although most patients who improved early maintained benefit, a small subgroup exhibited delayed improvement, while another subgroup experienced transient early response followed by loss of effect. Among patients who had not achieved ≥50% pain relief by Week 3, approximately half ultimately became 12-month responders. This subgroup suggests that the absence of early improvement, particularly within the first several weeks, should not immediately be interpreted as treatment failure. At the same time, classification in the present study was anchored at Week 6, which appeared to represent a more stable point for defining response patterns.

Conversely, approximately 9% of patients who initially met responder criteria early in the post-procedure period no longer met this threshold at 12 months, indicating that early improvement alone does not guarantee durable success. The subgroup of transient early responders may reflect several underlying mechanisms. One possibility is a nonspecific early treatment response, including placebo- or expectation-driven effects. Such responses have been well documented in sham-controlled BVNA and other spine procedure studies [[5], [6], [7], [8],[27], [28], [29], [30], [31]], in which a substantial proportion of sham-treated patients achieved clinically meaningful improvement. Alternatively, early improvement followed by loss of response may indicate the presence of multiple competing pain generators not fully addressed by BVNA.

These findings underscore the importance of longitudinal follow-up, as early improvement alone may not reliably identify likelihood of durable benefit. Taken together, reliance on a single early time point may be insufficient. Instead, serial evaluation of response trajectory may provide a more accurate and clinically meaningful indicator of long-term outcomes following BVNA.

### 4.5 Contextualizing early trajectory vs. baseline characteristics

Most baseline demographic and clinical characteristics were not independently associated with 12-month response. Age, sex, BMI, baseline pain severity, and lesion duration strategy did not meaningfully distinguish responders from nonresponders [28]. In contrast, early post-procedural response remained independently associated with long-term success after multivariable adjustment. This suggests that the observed biological response to treatment may be more informative than baseline characteristics alone.

Nonetheless, early response should not be interpreted in isolation; rather, the stability of response over time may provide a more reliable indicator of true treatment effect than a single early time point. This concept is clinically relevant because many baseline characteristics previously proposed in the BVNA literature have shown inconsistent results across studies [1,[5], [6], [7], [8], [9], [10], [11], [12], [13]]. Early trajectory plausibly reflects the combined influence of patient biology, endplate pathology, inflammatory state, healing response, and technical adequacy of ablation. As such, it may provide a more individualized and clinically relevant marker of treatment effect.

### 4.6 Prior treatment failure rather than procedure burden

The relationship between the number of treated levels and clinical outcome should be interpreted cautiously. In this cohort, the number of treated levels may not directly reflect underlying disease burden, as insurance constraints sometimes limited treatment to fewer vertebral levels despite the presence of additional Modic-positive segments. As a result, this variable may not accurately capture treatment adequacy, and its lack of association with 12-month outcomes should be interpreted with caution and within the context of treatment constraints.

The present study also suggests that prior treatment history may influence the likelihood of durable response, concordant with prior reports [24,25,27,28,32]. Patients with a history of failed prior spine-related procedures had lower odds of achieving 12-month benefit after BVNA. Regardless, exploratory analyses indicated that the total number of prior spine-related procedures was not independently associated with clinical outcomes. Thus, procedural burden alone does not appear to preclude meaningful benefit from BVNA. Rather, the pattern of repeated prior treatment failures may be a more clinically relevant marker of a complex or multifactorial pain phenotype. This distinction certainly has practical implications. For instance, patients who have undergone numerous prior spine-related interventions should not necessarily be excluded from consideration for BVNA solely on the basis of their procedural volumes. However, clinicians may wish to interpret repeated prior treatment failures as a signal to consider additional pain generators, overlapping pathology, or a lower probability of a durable response.

### 4.7 Functional recovery and reduced healthcare utilization

Early pain improvement corresponded to functional outcomes beyond pain scores. Patients achieving 50% response by Week 6 were more likely to achieve clinically meaningful ODI improvement at 12 months, suggesting a link between early analgesia and longer-term function. Similarly, subjective patient-reported improvement also paralleled these findings. The proportion of patients reporting meaningful global improvement increased over time and remained stable through 12 months, suggesting that these early gains were durable. The reductions in subsequent healthcare utilization further support the potential clinical implications of the study findings. Marked decreases in spine-related injections and other interventions after BVNA are consistent with a reduction in the need for ongoing procedural management. Although these observations do not establish causality, they suggest that early identification of likely responders and nonresponders may help guide more efficient and individualized follow-up care.

### 4.8 Potential biological explanations for response trajectories

The biological basis underlying the observed response trajectories remains uncertain and warrants further investigation. Delayed improvement may reflect the time required for resolution of vertebral endplate inflammation, modulation of nociceptive signaling, and remodeling of adjacent bone and endplate tissue following ablation [[15], [16], [17]]. Conversely, transient early improvement followed by loss of response may indicate that some patients have multiple concurrent pain generators beyond the vertebrogenic source targeted by BVNA. It is also possible that differing patterns of local inflammation, bone remodeling, or central sensitization may contribute to the variability in response to this procedure.

Future studies incorporating longitudinal imaging, inflammatory biomarkers, and repeated patient-reported outcomes may help clarify the biological mechanisms underlying treatment response. Such work may explain why some patients improve rapidly, others improve more gradually, and a minority fail to achieve durable benefit after BVNA.

### 4.9 Limitations and future directions

Several methodologic limitations should be considered, many of which are inherent to the study design. First, although this multicenter cohort included 140 patients with prospective follow-up through 12 months, the relatively small number of nonresponders (n = 25) limits the precision of estimates related to negative predictive performance and constrains the stability of multivariable modeling. As such, the observed effect sizes should be interpreted with appropriate caution. Second, the AUC thresholds identified in this study, including the approximately 35–40% pain relief at Week 6, should be interpreted as cohort-derived estimates that require confirmation in independent populations before clinical adoption. These thresholds may reflect model-specific optimization and may not generalize beyond the current sample. Nevertheless, the data suggest that early trajectory may provide a useful framework for counseling and follow-up.

Third, the overall responder rate in this cohort may limit discrimination between outcome groups and may inflate measures of model performance, including the area under the receiver operating characteristic curve. Accordingly, the reported discrimination should be interpreted in the context of outcome imbalance and requires validation in more balanced datasets. Fourth, although efforts were made to address confounding through multivariable adjustment and standardized data collection across sites, residual confounding inherent to an observational study design cannot be excluded. In particular, unmeasured clinical and procedural factors may additionally influence both early response trajectories and long-term outcomes. Fifth, trajectory classifications were descriptive and not intended for formal subgroup inference. Accordingly, these trajectory patterns should be interpreted as descriptive representations of response heterogeneity rather than as distinct clinical phenotypes. Finally, internal validation was not a focus of the current analysis, which was designed to evaluate associations rather than to develop a fully specified predictive model. As such, external validation in independent populations will be important to assess the reproducibility and generalizability of these findings.

Despite these limitations, this study provides prospective, high-frequency longitudinal data capturing early response trajectories following BVNA, extending beyond prior work that has relied on discrete follow-up intervals. These findings support the clinical relevance of tracking response patterns over time rather than relying solely on fixed outcome thresholds. Future studies should validate these observations in larger and more balanced multicenter cohorts. Longitudinally tracking response patterns would help to refine early-response thresholds and determine their role in guiding clinical reassessment strategies and optimize long-term outcomes.

## 5 Conclusion

This study extends prior work by moving beyond when improvement occurs after BVNA to address when early response becomes clinically actionable. Early patterns of improvement were associated with 12-month outcomes. Notably, submaximal improvement at an early time point identified patients who achieved durable, clinically meaningful benefit, whereas absence of early improvement was associated with a lower, but not negligible, probability of long-term response. These findings suggest that early response should not be interpreted in binary terms alone; rather, the degree and pattern of improvement may help guide clinical expectations, follow-up strategies, and shared decision-making.

## Funding

This work was supported in part by internal funding from the 10.13039/100007184Yale School of Medicine, 10.13039/100030726Department of Orthopaedics and Rehabilitation, New Haven, CT, USA.

## Declaration of competing interest

The authors report the following relationships that could be perceived as potential conflicts of interest. Dr. Charles Odonkor serves on the Research Committee of the International Pain and Spine Intervention Society and has received travel support from the organization. He has also provided consulting or advisory services to Boston Scientific and SPR Pain Relief. Drs. Jack Diep and Selaiman Noori are consultants for Boston Scientific and Abbott. Dr. David Lee is a consultant for Boston Scientific, Abbott, Mainstay Medical, WISE, Petal Surgical. The remaining authors declare no financial or personal relationships that could have influenced the work presented in this manuscript.
